# Hospital Patient Demographics and Administration of Intravenous Thrombolysis in Acute Ischemic Stroke

**DOI:** 10.1001/jamanetworkopen.2024.62271

**Published:** 2025-02-28

**Authors:** Jean-Luc K. Kabangu, Adip G. Bhargav, Delaney Graham, Amanda Hernandez, Sonia V. Eden

**Affiliations:** 1 Department of Neurological Surgery, University of Kansas Medical Center, Kansas City; 2 School of Medicine, University of Tennessee Health Science Center, Memphis; 3 School of Medicine, University of Michigan, Ann Arbor; 4 Department of Neurological Surgery, Semmes-Murphey Clinic, Memphis, Tennessee; 5 Department of Neurological Surgery, University of Tennessee Health Science Center, Memphis

## Abstract

**Importance:**

Stroke is a major cause of morbidity and mortality. Timely administration of intravenous thrombolysis (IVT) is essential for improving outcomes for patients with acute ischemic stroke. Significant disparities exist in IVT administration based on socioeconomic and racial and ethnic backgrounds. Understanding how hospital-level segregation is associated with stroke treatment outcomes is crucial for addressing these disparities.

**Objective:**

To investigate the association between hospital segregation, using the Index of Concentration at the Extremes (ICE), and IVT administration rates among patients with stroke.

**Design, Setting, and Participants:**

This retrospective cohort study used US hospital data from the 2016-2020 National Inpatient Sample database. Using *International Statistical Classification of Diseases and Related Health Problems, Tenth Revision, Clinical Modification* codes, patients admitted to hospitals with acute ischemic stroke were included. Statistical analysis was performed from March through July 2024.

**Exposure:**

Hospital segregation measured by the ICE, quantifying socioeconomic and racial and ethnic extremes within hospitals. ICE values range from −1 (predominantly Black and socioeconomically disadvantaged) to 1 (predominantly White and socioeconomically advantaged). Hospitals were categorized into ICE quintiles, with the first quintile representing the most disadvantaged hospitals, the third quintile representing a balanced patient mix, and the fifth quintile representing the most privileged hospitals.

**Main Outcomes and Measures:**

The primary outcome was IVT administration rates. Secondary outcomes included racial and ethnic disparities in IVT administration across ICE quintiles.

**Results:**

Among 2 494 945 patients with stroke, the mean (SD) age was 70.1 (14.0) years, 50.2% were male, 0.5% were American Indian, 3.1% were Asian or Pacific Islander, 17.4% were Black, 8.2% were Hispanic, 68.2% were White, and 2.6% were other race or ethnicity. Of these patients, 65.4% were treated at hospitals in the third ICE quintile, while 1.2% of patients were treated at hospitals in the first ICE quintile. Patients at hospitals in the fourth and fifth ICE quintiles were significantly more likely to receive IVT (fourth quintile: adjusted odds ratio [AOR], 1.32 [95% CI, 1.26-1.38]; fifth quintile: AOR, 1.27 [95% CI, 1.21-1.34]) compared with those in the first quintile. Racial and ethnic disparities in IVT administration were most pronounced in the first ICE quintile, where Black patients were 32% less likely than White patients to receive IVT (AOR, 0.68 [95% CI, 0.58-0.79]). This disparity decreased but persisted in higher quintiles.

**Conclusions and Relevance:**

In this study of hospital segregation and IVT administration rates, segregation was associated with lower likelihood of IVT administration for patients at hospitals serving predominantly Black and socioeconomically disadvantaged communities. Socioeconomic improvements were associated with reduced, but not eliminated, racial and ethnic disparities in stroke treatment. Addressing structural racism and segregation is crucial for equitable access to stroke care.

## Key Points

**Question:**

Is hospital segregation, as measured by the Index of Concentration at the Extremes, associated with rates of intravenous thrombolysis (IVT) use among patients with acute ischemic stroke?

**Findings:**

In this cohort study of 2 494 945 patients from the National Inpatient Sample database from 2016 to 2020, hospitals serving predominantly Black and socioeconomically disadvantaged communities had significantly lower IVT administration rates. Patients at these hospitals were less likely to receive IVT compared with those at more affluent hospitals.

**Meaning:**

This study suggests that hospital segregation was associated with IVT administration, highlighting the need to address structural racism and segregation to ensure equitable access to stroke care.

## Teaser

This cohort study uses data from the National Inpatient Sample database to investigate the association of hospital segregation with intravenous (IV) thrombolysis administration rates among patients with stroke.

## Introduction

Stroke is a leading cause of morbidity and mortality worldwide, with timely administration of intravenous thrombolysis (IVT) being crucial for improving outcomes for patients with acute ischemic stroke.^1,2^ Despite the clear benefits of IVT, significant disparities exist in its administration based on socioeconomic and racial and ethnic backgrounds.^2^ Prior studies have highlighted the role of structural racism and residential segregation in creating and perpetuating these health care disparities, yet there is limited understanding of how hospital-level segregation is associated with stroke treatment outcomes.^1,2,3^

The Index of Concentration at the Extremes (ICE) provides a measure to quantify socioeconomic and racial segregation, offering a unique perspective on the distribution of health care resources and services.^4,5^ Unlike previous studies, which largely focus on individual or residential factors, this study applies the ICE to hospital-level polarization to evaluate its association with disparities in IVT administration. By shifting the focus from patient-level determinants to structural hospital-level factors, this study introduces a novel framework for understanding inequities in stroke care.^1,2,3,4,5^

We hypothesize that patients treated at hospitals serving predominantly Black and socioeconomically disadvantaged communities will have lower rates of IVT administration compared with those treated at hospitals serving more socioeconomically advantaged communities. In addition, we examine whether hospitals with greater socioeconomic advantage show reduced disparities in IVT administration across racial and ethnic groups.

By exploring these associations, this study seeks to provide novel insights into the association between hospital segregation and stroke treatment disparities. Understanding these dynamics is essential for developing targeted interventions to improve health care equity and ensure that all patients, regardless of socioeconomic or racial and ethnic background, have access to timely and effective stroke care.

## Methods

### Data Source

This study was conducted in accordance with the Strengthening the Reporting of Observational Studies in Epidemiology (STROBE) reporting guideline for cohort studies. It used the National Inpatient Sample (NIS), a component of the Healthcare Cost and Utilization Project, which provides publicly available, deidentified, nationally representative inpatient data from US hospitals^6^; therefore, patient consent was not required. The NIS uses discharge weights from participating hospitals to provide nationally representative estimates. We identified patients admitted with a primary diagnosis of acute ischemic stroke from 2016 to 2020 using the *International Statistical Classification of Diseases and Related Health Problems, Tenth Revision, Clinical Modification* codes (eTable 1 in Supplement 1). The data comply with the Health Insurance Portability and Accountability Act regulations, ensuring that no individual patient can be identified. According to the guidelines of the Agency for Healthcare Research and Quality, which oversees the Healthcare Cost and Utilization Project, studies using the NIS database do not constitute human participants research. Therefore, institutional review board approval was not sought, as this research is exempt from the need for review or oversight.

### Primary Exposure, Measures, and Outcomes

The primary exposure was hospital polarization, measured by the ICE. The ICE is a measure used in social science and public health research to quantify the degree of segregation or inequality.^4,5^ It captures the distribution of a population across extreme categories, such as the most socioeconomically disadvantaged and the most socioeconomically advantaged groups.^4,5^ Although traditionally used to measure residential segregation, we adapted the formula to calculate segregation of patients with stroke within hospitals across the US. Specifically, we examined the concentrated extremes of racial and ethnic and socioeconomic privilege, defined as socioeconomically advantaged White patients with stroke, and deprivation, encompassing socioeconomically disadvantaged Black patients with stroke.

We calculated the ICE as follows: ICE*_i_* = (*A_i_* − *P_i_*/*T_i_*). In our study, *A_i_* represents the total number of White patients with stroke residing in zip codes within the top quartile of median household income, *P_i_* represents the total number of Black patients with stroke living in zip codes within the bottom quartile of median household income, and *T_i_* represents the total number of patients with stroke across all racial and ethnic groups and median household income levels treated at the *i*th hospital. The NIS provides racial and ethnic stratification, categorizing patients as American Indian (the NIS uses “Native American,” but we are using “American Indian” throughout), Asian or Pacific Islander, Black, Hispanic, White, and other. The “other” category includes patients whose race and ethnicity were recorded as a combination of multiple groups or other nonspecified categories. Information on race and ethnicity is often collected via self-report at the time of hospital admission, but reporting practices vary across hospitals and states.

ICE values, ranging from −1 to 1, indicate the socioeconomic and racial and ethnic composition of patients with stroke within hospitals. An ICE value close to 1 signifies hospitals serving predominantly White patients with stroke from the top quartile of median household income, while an ICE value close to −1 indicates those serving predominantly Black patients with stroke from the bottom quartile of median household income. An ICE value around 0 suggests a balanced mix of patients. Following the method of Zhang et al,^4^ ICE values were categorized into quintiles: the first quintile represents the group with the greatest level of deprivation (predominantly Black and socioeconomically disadvantaged patients); the second quintile includes fewer Black patients from socioeconomically disadvantaged neighborhoods; the middle quintile has a balanced mix; the fourth quintile has more White patients from socioeconomically advantaged neighborhoods; and the fifth quintile represents the most privileged group (predominantly White and socioeconomically advantaged patients). A sample calculation is provided in the eAppendix in Supplement 1.

Our primary outcome was the administration of IVT for acute ischemic stroke. Given that IVT must be administered within 4.5 hours of symptom onset, this outcome inherently reflects timely treatment. In the context of stroke care during the study period, alteplase was the only US Food and Drug Administration–approved thrombolytic agent for IVT; thus, all cases of IVT administration in this study are presumed to involve alteplase.^7^

Our secondary outcome examined differences in IVT administration by racial and ethnic groups across ICE quintiles. Patient comorbidities were derived using secondary discharge diagnoses codes from the *International Statistical Classification of Diseases and Related Health Problems, Tenth Revision* (eTable 1 in Supplement 1).

### Statistical Analysis

Statistical analysis was performed from March through July 2024. Logistic regression was used to assess the association between hospital ICE quintiles and IVT administration. Multivariable models were adjusted for patient age, sex, National Institute of Health Stroke Scale (NIHSS) score, primary expected payer (Medicare, Medicaid, private insurance, self-pay, no charge, and other), zip code median household income quartile, year, hospital characteristics (geographic location, rural-urban National Center for Health Statistics location, teaching status, and bed size), patient comorbidities (hypertension, diabetes, coronary artery disease, atrial fibrillation, chronic kidney disease, congestive heart failure, hyperlipidemia, and presence of intracerebral hemorrhage), and whether the patient was transferred from a different facility. Covariates were selected based on clinical relevance, prior literature, and statistical significance in univariate analyses, following established methods in studies on disparities in IVT use.^8^

ICE quintiles were used in the primary analysis to facilitate interpretation and comparison across distinct levels of hospital segregation, consistent with prior studies. Sensitivity analyses using ICE value as a continuous variable showed consistent results, supporting the robustness of the association between ICE value and IVT administration disparities.

We first investigated the differences in IVT use by ICE quintiles, using the first-quintile (most disadvantaged) hospitals as the reference. This analysis was then repeated by stratifying by race and ethnicity (Asian or Pacific Islander, Black, Hispanic, and White; American Indian patients were not included due to their small sample size in the NIS, which limited the feasibility of meaningful statistical analysis). Last, we compared IVT use between White patients with stroke (as the reference) and patients with stroke from other racial and ethnic groups (Asian or Pacific Islander, Black, and Hispanic; American Indian patients were not included due to their small sample size in the NIS, which limited the feasibility of meaningful statistical analysis) by ICE quintiles to assess potential ICE-associated variations in disparities. All significance tests were 2-sided, with *P* ≤ .05 defined as statistically significant. Statistical analysis was conducted using IBM SPSS, version 29 (IBM Corp) and Python, version 3.12.0 (Python Software Foundation).

## Results

A total of 2 494 945 patients with stroke (mean [SD] age, 70.1 [14.0] years; 50.2% men and 49.8% women; 0.5% American Indian patients, 3.1% Asian or Pacific Islander patients, 17.4% Black patients, 8.2% Hispanic patients, 68.2% White patients, and 2.6% patients of other race or ethnicity) met the inclusion criteria; exclusions are detailed in eFigure 1 in Supplement 1. Of these, 1 651 465 patients (65.4%) were treated at hospitals in the third ICE quintile. In contrast, 29 765 patients (1.2%) were treated at hospitals in the first quintile and 96 605 patients (3.8%) were treated at hospitals in the fifth quintile. Patients treated in first-quintile hospitals were younger than those treated in fifth-quintile hospitals (mean [SD], 65.9 [13.8] vs 73.8 [13.5] years) and had higher mean (SD) NIHSS scores (6.6 [6.7] vs 5.3 [6.5]) (Table 1). In addition, a greater proportion of patients treated in first-quintile hospitals than in fifth-quintile hospitals were self-pay (4.3% vs 1.6%) (eTable 2 in Supplement 1).

**Table 1.** Socioeconomic, Clinical, and Hospital Characteristics by ICE Quintiles^a^

Characteristic	ICE quintile, No. (%)					*P* value
	First	Second	Third	Fourth	Fifth	
Age, mean (SD), y	65.9 (13.8)	67.1 (13.9)	70.1 (14.0)	71.4 (14.1)	73.8 (13.5)	<.001
NIHSS score, mean (SD)	6.6 (6.7)	6.8 (7.1)	6.6 (7.2)	6.3 (7.2)	5.3 (6.5)	<.001
Zip code median household income quartile						
First (≤25th percentile)	26 115 (88.6)	167 395 (63.0)	525 190 (32.3)	32 475 (7.4)	1335 (1.4)	<.001
Second (26th-50th percentile)	1570 (5.3)	53 550 (20.2)	527 315 (32.5)	61 255 (13.9)	2915 (3.1)	
Third (51st-75th percentile)	1110 (3.8)	33 760 (12.7)	407 815 (25.1)	130 290 (29.5)	10 580 (11.1)	
Fourth (76th-100th percentile)	665 (2.3)	10 815 (4.1)	163 210 (10.1)	217 140 (49.2)	80 565 (84.5)	
Sex						
Male	14 275 (48.0)	134 315 (49.8)	831 245 (50.3)	224 025 (50.1)	47 550 (49.2)	<.001
Female	15 485 (52.0)	135 560 (50.2)	819 980 (49.7)	223 150 (49.9)	49 045 (50.8)	
Hospital teaching status						
Rural	5650 (19.0)	30 610 (11.3)	134 080 (8.1)	2045 (0.5)	395 (0.4)	<.001
Urban nonteaching	2890 (9.7)	29 010 (10.7)	337 525 (20.4)	97 920 (21.9)	32 000 (33.1)	
Urban teaching	21 225 (71.3)	210 285 (77.9)	1 179 860 (71.4)	347 240 (77.6)	64 210 (66.5)	
Number of hospital beds						
Small	9195 (30.9)	32 455 (12.0)	269 885 (16.3)	84 935 (19.0)	32 330 (33.5)	<.001
Medium	12 585 (42.3)	72 515 (26.9)	450 440 (27.3)	147 130 (32.9)	40 990 (42.4)	
Large	7985 (26.8)	164 935 (61.1)	931 141 (56.4)	215 140 (48.1)	23 285 (24.1)	
Patient race and ethnicity						
American Indian	40 (0.1)	850 (0.3)	8940 (0.5)	1400 (0.3)	125 (0.1)	<.001
Asian or Pacific Islander	175 (0.6)	2070 (0.8)	45 355 (2.8)	24 355 (5.6)	3775 (4.0)	
Black	23 515 (81.2)	126 055 (47.4)	226 110 (13.9)	46 370 (10.6)	5400 (5.7)	
Hispanic	1005 (3.5)	13 445 (5.1)	151 810 (9.3)	32 815 (7.5)	3180 (3.3)	
White	3600 (12.4)	118 345 (44.5)	1 151 730 (70.8)	319 070 (72.9)	80 885 (84.8)	
Other	615 (2.1)	5065 (1.9)	42 170 (2.6)	13 870 (3.2)	1990 (2.1)	
Comorbidities						
Diabetes	14 100 (47.4)	116 290 (43.1)	650 450 (39.4)	161 335 (36.1)	31 205 (32.3)	<.001
Hypertension	17 190 (57.8)	158 320 (58.7)	962 025 (58.3)	259 980 (58.1)	56 780 (58.8)	<.001
Hyperlipidemia	14 765 (49.6)	152 390 (56.5)	1 004 615 (60.8)	280 920 (62.8)	62 370 (64.6)	<.001
Chronic kidney disease	6260 (21.0)	50 015 (18.5)	301 730 (18.3)	81 840 (18.3)	17 160 (17.8)	<.001
Atrial fibrillation	4470 (15.0)	54 525 (20.2)	422 805 (25.6)	125 940 (28.2)	28 915 (29.9)	<.001
Congestive heart failure	6025 (20.2)	49 590 (18.4)	271 870 (16.5)	69 080 (15.4)	13 940 (14.4)	<.001
Coronary artery disease	6340 (21.3)	65 840 (24.4)	435 555 (26.4)	111 830 (25.0)	24 530 (25.4)	<.001
Intracerebral hemorrhage	650 (2.2)	10 280 (3.8)	69 850 (4.2)	20 110 (4.5)	3395 (3.5)	<.001

Abbreviations: ICE, Index of Concentration at the Extremes; NIHSS, National Institutes of Health Stroke Scale.

^a^ The ICE quintiles are defined as follows: first quintile (most deprived), second quintile, third quintile, fourth quintile, and fifth quintile (most privileged).

Compared with patients treated at hospitals in the first quintile (predominantly Black and socioeconomically disadvantaged), those treated at hospitals in the fourth quintile (moderately White and socioeconomically advantaged) had an adjusted odds ratio (AOR) of 1.32 (95% CI, 1.26-1.38; *P* < .001) for receiving IVT, and those treated at hospitals in the fifth quintile (predominantly White and socioeconomically advantaged) had an AOR of 1.27 (95% CI, 1.21-1.34; *P* < .001) for receiving IVT (Figure). Patients treated at hospitals in the second quintile (moderately Black and socioeconomically disadvantaged) had an AOR of 1.27 (95% CI, 1.21-1.32; *P* < .001), and those treated at hospitals in the third quintile (balanced race and income mix) had an AOR of 1.23 (95% CI, 1.17-1.29; *P* < .001). In a sensitivity analysis using ICE as a continuous variable, ICE was associated with IVT administration (AOR, 1.03 [95% CI, 1.01-1.04]; *P* = .007).

**Figure.**
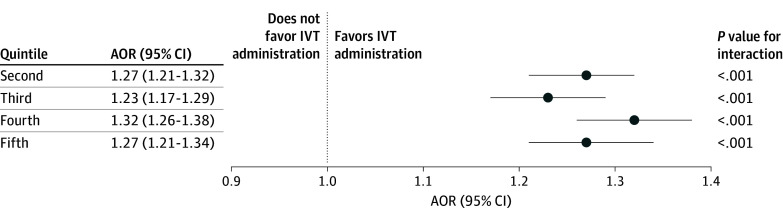
Adjusted Odds Ratios (AORs) for Intravenous Thrombolysis (IVT) Administration by Index of Concentration at the Extremes (ICE) Quintiles for Patients of All Racial and Ethnic Groups Hospitals were categorized into ICE quintiles, with the first quintile representing the most disadvantaged hospitals, the third quintile representing a balanced patient mix, and the fifth quintile representing the most privileged hospitals. Comparisons are made with first-quintile hospitals as the reference group.

White patients in fourth-quintile hospitals had an AOR of 1.39 (95% CI, 1.22-1.57; *P* < .001) for receiving IVT, and those in fifth-quintile hospitals had an AOR of 1.33 (95% CI, 1.17-1.52; *P* < .001) for receiving IVT, compared with those in the first quintile (Table 2). Black patients in fourth-quintile hospitals had an AOR of 1.25 (95% CI, 1.19-1.36; *P* < .001) for receiving IVT, and those in fifth-quintile hospitals had an AOR of 1.32 (95% CI, 1.19-1.48; *P* < .001) for receiving IVT, compared with those in first-quintile hospitals. Hispanic patients in fourth-quintile hospitals had an AOR of 1.45 (95% CI, 1.14-1.82; *P* = .002) for receiving IVT, and those in fifth-quintile hospitals had an AOR of 1.39 (95% CI, 1.08-1.80; *P* = .01) for receiving IVT, compared with those in first-quintile hospitals. No significant differences were observed for Asian or Pacific Islander patients between the first and other quintiles.

**Table 2.** Data on Intraracial Disparities in Intravenous Thrombolysis Administration by Socioeconomic and Racial Quintiles^a^

Characteristic	First ICE quintile, AOR (95% CI)	Second ICE quintile, AOR (95% CI)	*P* value	Third ICE quintile, AOR (95% CI)	*P* value	Fourth ICE quintile, AOR (95% CI)	*P* value	Fifth ICE quintile, AOR (95% CI)	*P* value
Race and ethnicity									
White	1 [Reference]	1.37 (1.21-1.56)	<.001^b^	1.34 (1.18-1.53)	<.001^b^	1.39 (1.22-1.57)	<.001^b^	1.33 (1.17-1.52)	<.001^b^
Black	1 [Reference]	1.25 (1.18-1.34)	<.001^b^	1.22 (1.15-1.29)	<.001^b^	1.25 (1.19-1.36)	<.001^b^	1.32 (1.19-1.48)	<.001^b^
Hispanic	1 [Reference]	1.22 (0.97-1.55)	.09	1.26 (1.00-1.59)	.04^b^	1.45 (1.14-1.82)	.002^b^	1.39 (1.08-1.80)	.01^b^
Asian or Pacific Islander	1 [Reference]	1.46 (0.76-2.82)	.26	1.48 (0.78-2.82)	.23	1.60 (0.84-3.05)	.15	1.35 (0.70-2.59)	.37

Abbreviations: AOR, adjusted odds ratio; ICE, Index of Concentration at the Extremes.

^a^ The ICE quintiles are defined as follows: first quintile (most deprived), second quintile, third quintile, fourth quintile, and fifth quintile (most privileged).

^b^ Statistical significance of the comparisons, with *P* ≤ .05 considered statistically significant.

The disparities in IVT administration between Black and White patients were greatest in the first quintile, where Black patients had an AOR of 0.68 (95% CI, 0.58-0.79; *P* < .001) compared with White patients (Table 3). This disparity among Black patients decreased across higher quintiles: AOR of 0.78 (95% CI, 0.76-0.80; *P* < .001) in the second quintile, AOR of 0.83 (95% CI, 0.80-0.85; *P* < .001) in the third quintile, and AOR of 0.86 (95% CI, 0.81-0.94; *P* = .001) in the fifth quintile. A similar trend was observed for Hispanic patients compared with White patients. Hispanic patients had an AOR of 0.68 (95% CI, 0.52-0.89; *P* = .005) in the first quintile, an AOR of 0.95 (95% CI, 0.93-0.96; *P* < .001) in the third quintile, an AOR of 1.05 (95% CI, 1.02-1.09; *P* = .003) in the fourth quintile, and no statistically significant difference in the fifth quintile (AOR, 1.06 [95% CI, 0.95-1.19]; *P* = .29). Table 3 shows that no significant disparity was observed between Asian or Pacific Islander patients and White patients in the first quintile (AOR, 0.56 [95% CI, 0.29-1.08]; *P* = .08). However, Asian or Pacific Islander patients in the second quintile faced greater disparities in IVT administration compared with those in the third, fourth, and fifth quintiles. Unadjusted analysis of intraracial and interracial disparities in IVT administration by socioeconomic and racial quintiles is detailed in eTable 3 in Supplement 1.

**Table 3.** Data on Interracial Disparities in Intravenous Thrombolysis Administration by Socioeconomic and Racial Quintiles

Characteristic	White, AOR (95% CI)	Black, AOR (95% CI)	*P* value	Hispanic, AOR (95% CI)	*P* value	Asian or Pacific Islander, AOR (95% CI)	*P* value
ICE quintile^a^							
First	1 [Reference]	0.68 (0.58-0.79)	<.001^b^	0.68 (0.52-0.89)	.005^b^	0.56 (0.29-1.08)	.08
Second	1 [Reference]	0.78 (0.76-0.80)	<.001^b^	0.80 (0.75-0.84)	<.001^b^	0.67 (0.58-0.77)	<.001^b^
Third	1 [Reference]	0.83 (0.82-0.84)	<.001^b^	0.95 (0.93-0.96)	<.001^b^	0.82 (0.80-0.85)	<.001^b^
Fourth	1 [Reference]	0.82 (0.80-0.85)	<.001^b^	1.05 (1.02-1.09)	.003^b^	0.92 (0.88-0.95)	<.001^b^
Fifth	1 [Reference]	0.86 (0.81-0.94)	<.001^b^	1.06 (0.95-1.19)	.29	0.85 (0.76-0.95)	.003^b^

Abbreviations: AOR, adjusted odds ratio; ICE, Index of Concentration at the Extremes.

^a^ The ICE quintiles are defined as follows: first quintile (most deprived), second quintile, third quintile, fourth quintile, and fifth quintile (most privileged).

^b^ Statistical significance of the comparisons, with *P* ≤ .05 considered statistically significant.

## Discussion

Our study highlights significant disparities in the administration of IVT among patients with stroke across different socioeconomic and racial and ethnic backgrounds, as measured by the ICE. By using the ICE to categorize hospitals based on the extremes of race and ethnicity and income, we were able to identify and quantify the association of socioeconomic and racial segregation with stroke treatment outcomes. To our knowledge, this study is the first to apply the ICE to measure segregation within hospitals and to demonstrate its association with IVT administration rates, providing a novel approach to understanding health care disparities.

The findings reveal a disparity in IVT administration rates. Patients in hospitals serving predominantly Black and socioeconomically disadvantaged communities (first quintile) were significantly less likely to receive IVT compared with those in hospitals serving predominantly White and socioeconomically advantaged patients (fifth quintile). This finding mirrors the broader trend observed in a study using ICE quintiles to explore the role of residential segregation in cancer outcomes, which found higher cancer mortality rates in more segregated, economically disadvantaged counties.^5^ Both situations underscore the profound association of structural inequalities with health outcomes.

Our results also show that racial and ethnic disparities persist within each ICE quintile. Black and Hispanic patients consistently received IVT less frequently than White patients, even within the same socioeconomic contexts. This finding indicates that factors beyond hospital resources and geographic location, such as implicit bias, differences in patient-clinician communication, and health literacy, are also associated with these disparities.^2^ For instance, Black patients are more likely to drive to the emergency department rather than take an ambulance, largely due to the fear of surprise ambulance bills, which can be substantial and often are not covered by insurance.^2,3,9,10^ This mode of arrival results in significant delays, affecting their eligibility for IVT, which must be administered within a 4.5-hour window from symptom onset. Once in the emergency department, Black patients frequently encounter longer door-to-needle times compared with their White counterparts, partly due to longer emergency department wait times and a higher likelihood of presenting to hospitals without formalized stroke care pathways.^1,11^ This lack of certification and structured pathways is associated with the lower likelihood of timely IVT administration, which helps explain our finding that White, Black, and Hispanic patients treated at first-quintile hospitals were less likely to receive IVT than those treated at fourth- and fifth-quintile hospitals.

Although the racial and ethnic disparity in IVT administration is most pronounced in the first quintile, where Black patients were 32% less likely to receive IVT compared with White patients, it gradually diminished but persisted in higher quintiles. For instance, in the fifth quintile, Black patients were still 15% less likely to receive IVT than White patients, indicating that socioeconomic improvements are associated with reduced, but not eliminated disparities. Structural racism, exemplified by practices such as redlining, has had lasting effects on community resources, including health care facilities.^2,12,13^ Redlined neighborhoods often have underfunded hospitals, contributing to poorer health outcomes.^14^ In addition, census tracts in the lowest ICE quintile have significantly slower greenspace growth compared with the highest ICE quintile, reflecting ongoing resource deprivation.^15^ The geographic clustering of patients of racial and ethnic minority populations in certain underresourced hospitals, often referred to as *minority-serving hospitals*, highlights the systemic nature of these disparities.^2^ These hospitals are typically underfunded and struggle to provide the same level of care as more affluent institutions, particularly in regions with high concentrations of racial and ethnic minorities and low-income populations, such as the South Atlantic region of the US.^16,17^ Studies show that enhancing hospital infrastructure in these disadvantaged areas can reduce health care disparities, addressing both historical and current inequities.^2^ Even with improved economic circumstances, Black patients still experience health care disparities. This finding is supported by a study on racial and ethnic and socioeconomic disparities in aneurysmal subarachnoid hemorrhage care, which found that patients from racial and ethnic minority groups, even from wealthier backgrounds, continue to face significant disparities in treatment and outcomes compared with White patients.^18^ The persistence of disparities among Black patients in more advantaged hospitals suggests that factors beyond economics, such as language barriers, cultural differences, and implicit biases in care delivery, are associated with these inequities, as depicted in eFigure 2 in Supplement 1.^9,18^

Segregation in hospitals is closely associated with residential segregation.^16^ The literature indicates that racial and ethnic and socioeconomic segregation is increasing across several sectors in the US, including schools. For instance, a study conducted by Stanford University found that segregation between White and Black students has increased by 64% since 1988 in the 100 largest school districts, and segregation by economic status has increased by 50% since 1991.^19^ Our findings support the evidence of the association between residential and hospital segregation.^16^ To address these disparities, increased funding to prevent hospital closures, support the training and advancement of Black physicians, and implement need-based reimbursement models are crucial.^20,21,22^ In addition, the isolation index, which measures the overall proportion of Black residents in an area, has been used to demonstrate that historical patterns of Black migration may have a strong intergenerational association with hospital segregation.^16^ Similarly, intergenerational poverty has been linked with increased stroke mortality rates, highlighting the profound long-term associations of socioeconomic disadvantages with health outcomes.^23^ This finding emphasizes the need for future research to incorporate historical location-based policies and structures into the study of hospital segregation. Moreover, variations in racial and ethnic segregation across US hospital markets may be associated with inequitable outcomes from health reform efforts, highlighting the importance of integrating equity measures into payment reform efforts to improve health care equity.^16^

To conceptualize the association of hospital segregation with IVT use, we propose a framework that integrates structural, systemic, and patient-level factors. Structural factors include disparities in hospital funding, infrastructure, and resource allocation, which are often exacerbated in minority-serving hospitals (underresourced hospitals with a higher proportion of patients from racial and ethnic minority groups). Systemic factors involve implicit biases, inequities in patient-clinician communication, and variability in hospital adherence to stroke care pathways. Patient-level factors, such as differences in transportation, health literacy, and stroke severity, further compound these disparities. This framework highlights how these interrelated elements are collectively associated with reduced IVT administration in segregated hospitals (eFigure 2 in Supplement 1).

Our study contributes to the existing body of literature by demonstrating that racial and ethnic segregation in health care delivery is persistent, despite the elimination of formal practices of racial and ethnic segregation decades ago. The consistent association of segregation with racial and ethnic disparities in health outcomes underscores the need for systemic interventions. Future health reform efforts must consider the broader context of hospital segregation to effectively address and mitigate health care disparities.

### Limitations

This study has several limitations that should be considered. First, the use of the NIS database, while comprehensive, relies on administrative data, which may be subject to coding errors and inaccuracies. Second, the ICE calculation relies on hospital data to approximate socioeconomic and racial and ethnic segregation, which may not fully capture the complexities of patient experiences and broader societal segregation. Third, although we adjusted for multiple covariates, there may be unmeasured confounding factors that could be associated with the outcomes. Fourth, the retrospective nature of the study limits our ability to establish causal relationships between hospital segregation and IVT administration rates. Fifth, our findings may not be generalizable to non-US health care settings or to hospitals not included in the NIS database. Future research should aim to address these limitations by using prospective data and incorporating more detailed measures of hospital resources and patient-level factors. In addition, multi-institutional studies that include patient-reported outcomes and surveys could help validate these findings and investigate the potential causes of this disparity.

## Conclusions

Our cohort study highlights significant disparities in IVT administration among patients with stroke based on socioeconomic and racial and ethnic backgrounds, as measured by the ICE. Patients at hospitals serving predominantly Black and socioeconomically disadvantaged communities are less likely to receive IVT compared with those at more affluent hospitals. In addition, higher ICE quintiles show reduced disparities in IVT administration across racial and ethnic groups. These findings underscore the association of structural racism and hospital segregation with health care outcomes. Addressing these fundamental issues is crucial for developing strategies that ensure equitable access to stroke care for all communities.

## Notes

Supplement 1. **eTable 1.** ICD-10-CM Codes for Variables Not Translated in the NIS **eAppendix.** Calculation of the Index of Concentration at the Extremes (ICE) **eFigure 1.** Cohort Selection Process for Stroke Patients (2016-2020) **eTable 2.** Socioeconomic, Clinical and Hospital Characteristics by ICE Quintiles Continued **eTable 3.** Unadjusted Odds Ratios (OR) and 95% Confidence Intervals (CI) for Intra-Race and Inter-Race Disparities in IVT Administration by Socioeconomic and Racial Quintiles **eFigure 2.** Potential Factors Affecting IVT Rates in Segregated Hospitals

Supplement 2.
Data Sharing Statement
