# Supplementary material for: Hospital Patient Demographics and Administration of Intravenous Thrombolysis in Acute Ischemic Stroke
Source: JAMA Netw Open. 2025 Feb 28;8(2):e2462271. doi: 10.1001/jamanetworkopen.2024.62271 (PMC11871536; doi:10.1001/jamanetworkopen.2024.62271)
Supplement: Supplement 1. — eTable 1. ICD-10-CM Codes for Variables Not Translated in the NIS eAppendix. Calculation of the Index of Concentration at the Extremes (ICE) eFigure 1. Cohort Selection Process for Stroke Patients (2016-2020) eTable 2. Socioeconomic, Clinical and Hospital Characteristics by ICE Quintiles Continued eTable 3. Unadjusted Odds Ratios (OR) and 95% Confidence Intervals (CI) for Intra-Race and Inter-Race Disparities in IVT Administration by Socioeconomic and Racial Quintiles eFigure 2. Potential Factors Affecting IVT Rates in Segregated Hospitals [file jamanetwopen-e2462271-s001.pdf]

Supplementary Online Content

Kabangu JLK, Bhargav AG, Graham D, Hernandez A, Eden SV. Hospital patient demographics and administration of intravenous thrombolysis in acute ischemic stroke. *JAMA Netw Open.* 2025;8(2):e2462271. doi:10.1001/jamanetworkopen.2024.62271

eTable 1. ICD-10-CM Codes for Variables Not Translated in the NIS

eAppendix. Calculation of the Index of Concentration at the Extremes (ICE)

eFigure 1. Cohort Selection Process for Stroke Patients (2016-2020)

eTable 2. Socioeconomic, Clinical and Hospital Characteristics by ICE Quintiles

Continued

eTable 3. Unadjusted Odds Ratios (OR) and 95% Confidence Intervals (CI) for Intra-Race and Inter-Race Disparities in IVT Administration by Socioeconomic and Racial Quintiles

eFigure 2. Potential Factors Affecting IVT Rates in Segregated Hospitals

This supplementary material has been provided by the authors to give readers additional information about their work.

© 2025 Kabangu JLK et al. *JAMA Network Open.*

eTable 1. ICD-10-CM Codes for Variables Not Translated in the NIS

**Variable**	**ICD – 10 - CM Code**
Acute Ischemic Stroke	163.0 - 163.9
Hypertension	I10-I15
Diabetes Mellitus	E10.618, E10.620 - E10.628, E10.65, E10.630, E10.69, E10.9, E11.618, E11.620-E11.628, E11.638, E11.00, E11.01, E11.630, E11.649, E11.65, E11.69, E11.9
Obesity	E66.01, E66.09, E66.1 - E66.9
Congestive Heart Failure	I11.0, I50.1, I50.9, I51.7
Alcohol Abuse	F10.10
Hyperlipemia	E78.0, E78.2, E78.4, E78.5
Smoking	Z87.891, F17.200
Coronary artery disease	I25.10
Arterial Fibrillation	I48.0 - I48.2, I48.91
Chronic Kidney Disease	N18.1 - N18.9

© 2025 Kabangu JLK et al. *JAMA Network Open.*

eAppendix. Calculation of the Index of Concentration at the Extremes (ICE)

To calculate the Index of Concentration at the Extremes (ICE) for our study, we used the following formula:

$$ICE_i = (A_i - P_i)/T_i$$

Where:

- A_i :The number of White stroke patients residing in the top quartile of median household income at hospital i.

- P_i :The number of Black stroke patients residing in the bottom quartile of median household income at hospital i.

- T_i: The total number of stroke patients (across all races and median household incomes) treated at hospital i.

Example Calculation

Consider Hospital i with the following data:

- A_i :150 White stroke patients living in zip codes in the top quartile of median household income.

- P_i : 50 Black stroke patients living in zip codes in the bottom quartile of median household income.

- T_i: 300 total stroke patients from all median household income zip codes.

The ICE for Hospital i is calculated as follows:

$$ICE_i = (150 - 50) / 300 = 0.33$$

Interpretation of ICE Values:

The ICE ranges from -1 to 1, representing the concentration of patients at the extremes of privilege and deprivation within a hospital, based on their residence in zip codes with median household incomes.

- ICE = 1: Indicates that all stroke patients in the hospital reside in zip codes with median household incomes in the top quartile and are White, representing the most privileged group.

© 2025 Kabangu JLK et al. *JAMA Network Open.*

- ICE = -1: Indicates that all stroke patients in the hospital reside in zip codes with median household incomes in the bottom quartile and are Black, representing the most deprived group.

- ICE = 0: Indicates an equal number of privileged and deprived patients, suggesting no concentration towards either extreme.

© 2025 Kabangu JLK et al. *JAMA Network Open.*

eFigure 1. Cohort Selection Process for Stroke Patients (2016-2020)

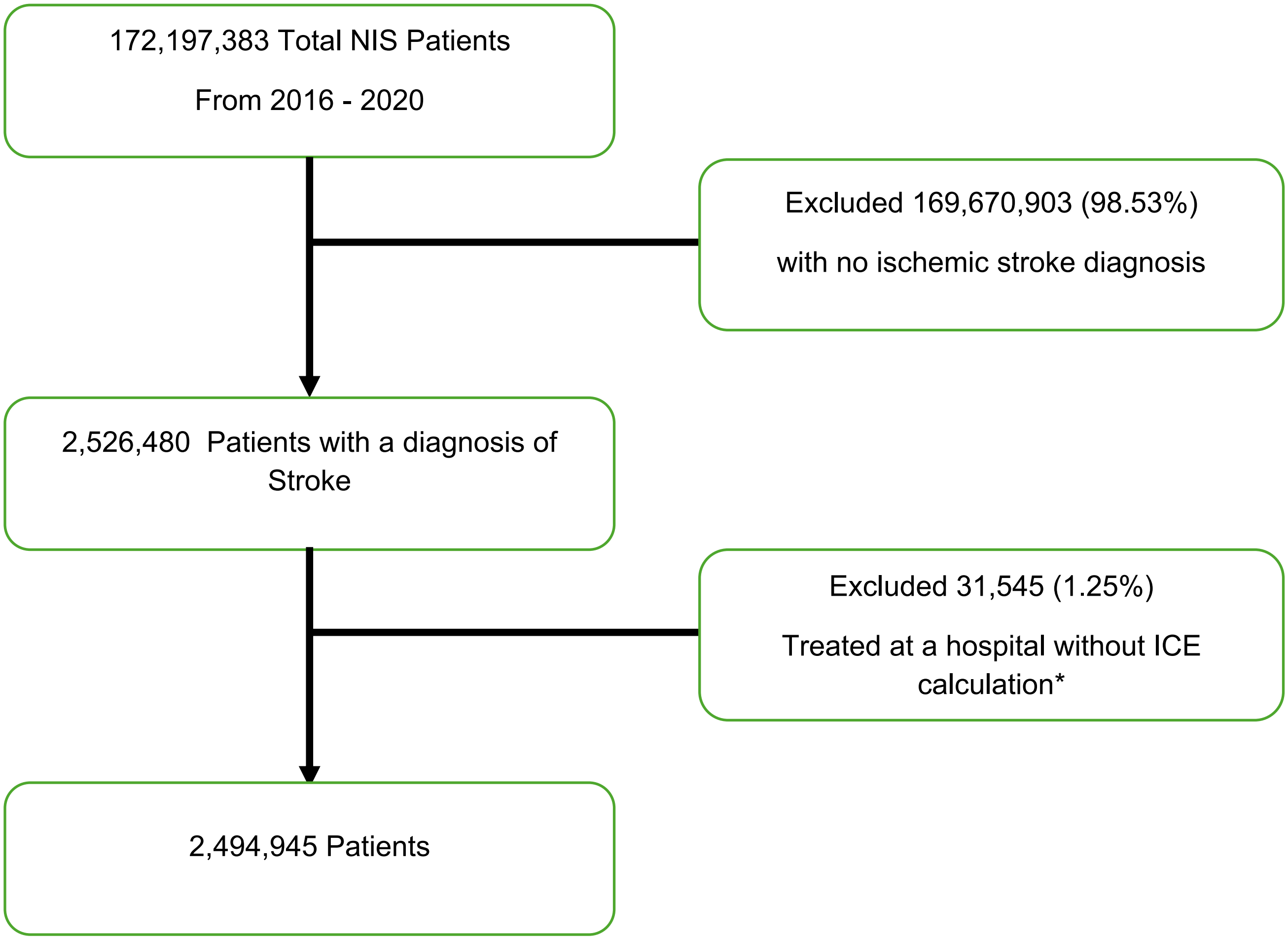

*Hospitals without an ICE value were those that did not treat either a Black patient from the lowest quartile of median household income zip codes or a White patient from the highest quartile, thereby preventing the calculation of ICE

© 2025 Kabangu JLK et al. *JAMA Network Open.*

eTable 2. Socioeconomic, Clinical and Hospital Characteristics by ICE Quintiles Continued						
Quintile	First	Second	Third	Fourth	Fifth	
	n (%)	n (%)	n (%)	n (%)	n (%)	*P*
**NCHS Urban/Rural Location**						**<0.001**
"Central" counties of metro areas of >=1 million population	19470 (65.5)	86820 (32.3)	418185 (25.4)	156755 (35.2)	17905 (18.6)	
"Fringe" counties of metro areas of >=1 million population	2230 (7.5)	36845 (13.7)	298105 (18.1)	197305 (44.3)	68585 (71.2)	
Counties in metro areas of 250,000-999,999 population	1255 (4.2)	54375 (20.2)	409060 (24.9)	58770 (13.2)	7550 (7.8)	
Counties in metro areas of 50,000-249,999 population	595 (2.0)	28800 (10.7)	199740 (12.1)	11950 (2.7)	805 (0.8)	
Micropolitan counties	3745 (12.6)	33740 (12.5)	186055 (11.3)	11460 (2.6)	1040 (1.1)	
Not metropolitan or micropolitan counties	2415 (8.1)	28460 (10.6)	134915 (8.2)	9115 (2.0)	375 (0.4)	
**Transfer In**						**<0.001**
Not transferred in or newborn admission	28165 (94.7)	232190 (86.5)	1393550 (84.7)	381045 (85.4)	89005 (92.3)	
Transferred in from a different acute care hospital	780 (2.6)	28585 (10.7)	200370 (12.2)	48240 (10.8)	3275 (3.4)	
Transferred in from another type of health facility	800 (2.7)	7510 (2.8)	51200 (3.1)	16730 (3.8)	4170 (4.3)	
**Census Division**						**<0.001**
New England	0 (0.0)	55 (0.0)	38035 (2.3)	55395 (12.4)	16165 (16.7)	
Mid-Atlantic	6235 (20.9)	17610 (6.5)	204390 (12.4)	71155 (15.9)	33600 (34.8)	
East North Central	10775 (36.2)	39440 (14.6)	262645 (15.9)	56050 (12.5)	9240 (9.6)	
West North Central	95 (0.3)	9510 (3.5)	102780 (6.2)	33930 (7.6)	3135 (3.2)	
South Atlantic	6370 (21.4)	106285 (39.4)	384775 (23.3)	56525 (12.6)	11410 (11.8)	
East South Central	3860 (13.0)	53125 (19.7)	138215 (8.4)	7760 (1.7)	1000 (1.0)	
West South Central	2015 (6.8)	38930 (14.4)	214295 (13.0)	30950 (6.9)	1460 (1.5)	
Mountain	0 (0.0)	185 (0.1)	102330 (6.2)	27965 (6.3)	4540 (4.7)	
Pacific	415 (1.4)	4765 (1.8)	204000 (12.4)	107475 (24.0)	16055 (16.6)	
**Primary Payer**						**<0.001**
Medicaid	17030 (57.3)	157120 (58.3)	1084645 (65.8)	295820 (66.2)	68030 (70.5)	
Medicare	6290 (21.2)	33510 (12.4)	154480 (9.4)	33260 (7.4)	4730 (4.9)	
Private Insurance	4550 (15.3)	51000 (18.9)	301230 (18.3)	96025 (21.5)	20770 (21.5)	
Self-Pay	1285 (4.3)	19285 (7.2)	65230 (4.0)	12735 (2.9)	1575 (1.6)	
No Charge	210 (0.7)	1570 (0.6)	4640 (0.3)	745 (0.2)	190 (0.2)	
Other	370 (1.2)	7005 (2.6)	38895 (2.4)	8240 (1.8)	1255 (1.3)	

© 2025 Kabangu JLK et al. *JAMA Network Open.*

**eTable 3.** Unadjusted Odds Ratios (OR) and 95% Confidence Intervals (CI) for Intra-Race and Inter-Race Disparities in IVT Administration by Socioeconomic and Racial Quintiles									
OR (95%CI) comparison by quintile									
**Race/ethnicity**	First quintile	Second quintile	*P-value*	Third quintile	*P-value*	Fourth quintile	*P-value*	Fifth quintile	*P-value*
White	1 (ref)	2.00 (1.76-2.26)	**<0.001**	1.85 (1.63-2.09)	**<0.001**	2.00 (1.91-2.09)	**<0.001**	1.57 (1.50-1.65)	**<0.001**
Black	1 (ref)	1.61 (1.53-1.71)	**<0.001**	1.85 (1.63-2.09)	**<0.001**	1.63 (1.54-1.72)	**<0.001**	1.34 (1.21-1.49)	**<0.001**
Hispanic	1 (ref)	1.55 (1.23-1.96)	**<0.001**	1.70 (1.35-2.14)	**<0.001**	2.00 (1.59-2.52)	**<0.001**	1.78 (1.39-2.29)	**<0.001**
Asian or PI	1 (ref)	1.46 (0.76-2.82)	**0.04**	2.21 (1.17-4.18)	**0.02**	2.57 (1.36-4.88)	**0.004**	2.09 (1.10-4.00)	**0.03**
All races/ethnic groups	1 (ref)	1.96 (1.02-3.77)	**<0.001**	1.82 (1.75-1.91)	**<0.001**	2.00 (1.91-2.09)	**<0.001**	1.57 (1.50-1.65)	**<0.001**
OR (95%CI) comparison by race/ethnicity									
**Quintile**	White	Black	*P-value*	Hispanic	*P-value*	Asian	*P-value*		
First	1 (ref)	0.95 (0.83-1.08)	0.43	1.09 (.84-1.41)	0.52	0.76 (0.40-1.46)	0.41		
Second	1 (ref)	0.77 (0.75-0.79)	**<0.001**	0.85 (0.80-0.89)	**<0.001**	0.75 (0.65-0.86)	**<0.001**		
Third	1 (ref)	0.86 (0.84-0.87)	**<0.001**	1.00(0.9901.02)	0.96	0.91 (0.89-0.94)	**<0.001**		
Fourth	1 (ref)	0.76 (0.74-0.79)	**<0.001**	1.08 (1.04-1.11)	**<0.001**	0.97 (0.93-1.01)	0.13		
Fifth	1 (ref)	0.82 (0.75-0.90)	**<0.001**	1.25 (1.13-1.39)	**<0.001**	1.03 (0.93-1.14)	0.58		

© 2025 Kabangu JLK et al. *JAMA Network Open.*

eFigure 2

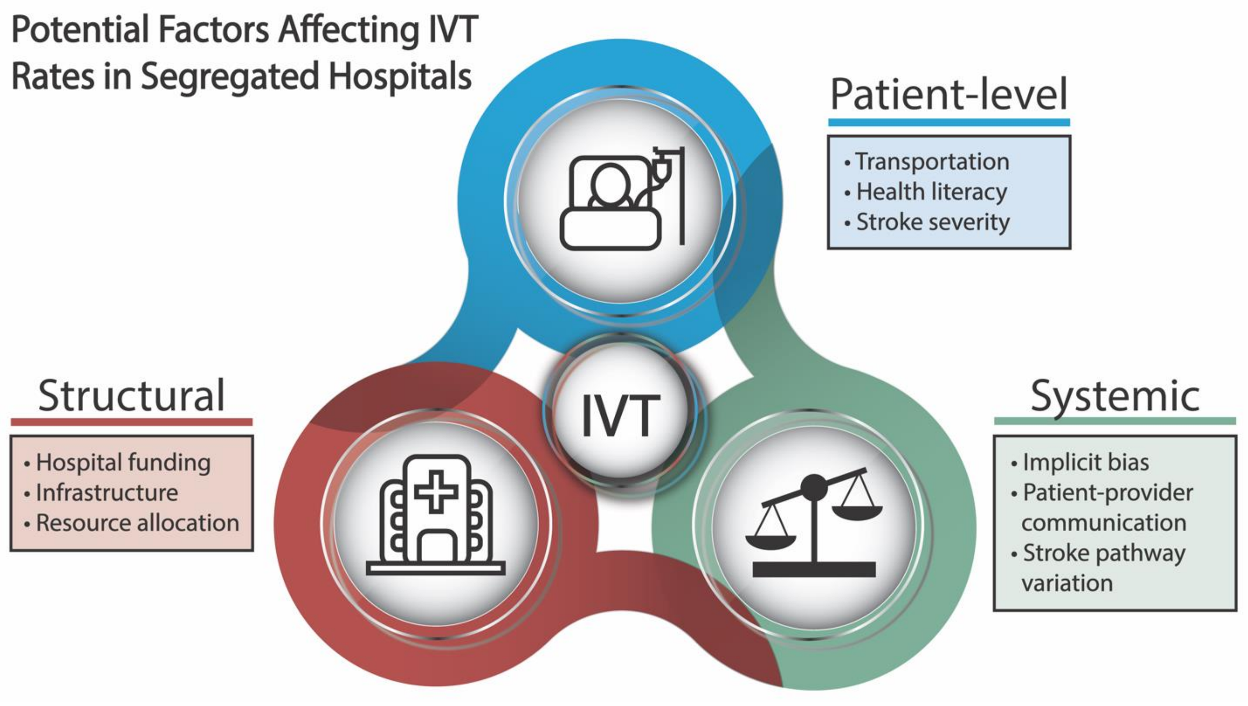

© 2025 Kabangu JLK et al. *JAMA Network Open.*
