# Supplementary material for: Hospital Patient Demographics and Administration of Intravenous Thrombolysis in Acute Ischemic Stroke
Source: JAMA Netw Open. 2025 Feb 28;8(2):e2462271. doi: 10.1001/jamanetworkopen.2024.62271 (PMC11871536; doi:10.1001/jamanetworkopen.2024.62271)
Supplement: Supplement 2. — Data Sharing Statement [file jamanetwopen-e2462271-s002.pdf]

Data Sharing Statement

Kabangu. Hospital Patient Demographics and Administration of Intravenous Thrombolysis in Acute Ischemic Stroke. *JAMA Netw Open*. Published February 28, 2025.
doi:10.1001/jamanetworkopen.2024.62271

Data
Data available: No
